# Clinico-epidemiological analysis of Post kala-azar dermal leishmaniasis (PKDL) cases in India over last two decades: a hospital based retrospective study

**DOI:** 10.1186/s12889-015-2424-8

**Published:** 2015-10-26

**Authors:** V. Ramesh, Himanshu Kaushal, Ashwani Kumar Mishra, Ruchi Singh, Poonam Salotra

**Affiliations:** Department of Dermatology, VMMC & Safdarjung Hospital, New Delhi, 110029 India; National Institute of Pathology (ICMR), Safdarjung Hospital Campus, New Delhi, 110029 India; NDDTC and Department of Psychiatry, AIIMS, New Delhi, 110029 India

**Keywords:** Diagnosis, Clinical Epidemiology, PKDL, Post kala-azar dermal leishmaniasis, Treatment, Visceral Leishmaniasis, Bihar

## Abstract

**Background:**

Patients with Post kala-azar dermal leishmaniasis (PKDL) are considered a reservoir of *Leishmania donovani.* It is imperative to identify and treat them early for control of visceral leishmaniasis (VL), a current priority in the Indian subcontinent. We explored trends in clinico-epidemiological features of PKDL cases over last two decades, for improving management of the disease.

**Methods:**

Clinically suspected cases were diagnosed with rK39 strip test followed by parasitological confirmation by microscopy and/or PCR/qPCR in skin tissue/slit aspirates. Patients were treated with antimonials till 2008 and subsequently with miltefosine.

**Results:**

The study indicated higher incidence of PKDL cases in areas of high endemicity for VL, with 20 % cases reporting no history of VL. Approximately 26 % cases of PKDL were initially misdiagnosed at primary health centers. Duration between onset of PKDL and diagnosis was above 12 months in 80 % cases. Diagnostic sensitivity was 32-36 % with microscopy and 96–100 % with PCR/qPCR. Compliance to treatment was over 85 % with miltefosine while 15 % with antimonials. Relapse rate with miltefosine was up to 13.2 %.

**Conclusions:**

PKDL patients tend to delay reporting and are often misdiagnosed. Confirmatory diagnosis using minimally invasive skin slit aspirate samples would help overcome such issues. There was a paradigm shift in compliance with miltefosine; however, increasing relapse rate indicated the need for newer therapies with oral formulations.

## Background

Leishmaniasis is an important parasitic disease, largely affecting poorest of the poor in developing countries, with over 350 million people worldwide considered at risk to the disease [1]. The disease manifests in different clinical forms, ranging from cutaneous leishmaniasis (CL) to disfiguring mucosal lesions to the most severe form, visceral leishmaniasis (VL). Currently, over 90 % of annual incidence of VL occurs in countries like India, Sudan, Bangladesh, Nepal, Brazil and Ethiopia [2]. Post kala-azar dermal leishmaniasis (PKDL), a dermal sequel of VL, is reported in areas endemic for *Leishmania donovani* in the Indian subcontinent, Sudan and its adjoining areas [3, 4], although sporadic cases have been reported from China, Japan, Iran and Iraq [5, 6]. In the Indian subcontinent, up to 15 % of apparently cured VL patients develop PKDL, as against 50–60 % in Sudan [3, 4, 7]. The disease is characterized by different clinical presentations from simple hypo-pigmented macular form to more developed lesions comprising of papular, nodular cutaneous lesions and/or polymorphic forms with mixed lesions. PKDL is not a life threatening disease, but more of a social stigma especially when lesions present on the exposed parts of the body. Importantly, in the Indian subcontinent, as the transmission of VL is anthroponotic, unattended cases of active PKDL serve as durable reservoir of *L. donovani* in VL endemic areas, especially during inter-epidemic period [1]. Hence it calls for better management of the disease. However, the clinical diagnosis of PKDL still remains perplexing and often requires an experienced clinician. Immunological diagnosis using rK39 strip test is not reliable for PKDL since antibodies could be persisting due to past episode of VL. Confirmatory diagnosis with microscopy has limited sensitivity and molecular techniques such as PCR/qPCR are available only in limited centers.

For the past several decades, pentavalent antimonials had been the first line treatment for management of VL and amphotericin B deoxycholate and pentamidine remained as second-line therapies. During 1980s, VL cases from Bihar, India developed resistance to pentavalent antimonials and isolates of *Leishmania* were found resistant to the drug [8, 9]. As a result, several formulations such as amphotericin B, miltefosine and paromomycin have been approved for treatment in the last decade. Initially, it was considered that VL cases treated with sodium antimony gluconate (SAG) may have higher probability of developing PKDL [10], however, subsequently several cases of PKDL were reported following VL treatment with other anti-leishmanial drugs including miltefosine and amphotericin B [11, 12]. The number of cases following treatment with drugs other than SAG may go up, as PKDL develops after several years of cure from VL [13]. Therefore, a greater insight into various clinical and epidemiologic aspects of PKDL is warranted. The present study explored trends in clinical and epidemiological features of PKDL cases over last two decades in a tertiary hospital setting, constituting the largest study undertaken so far in Indian PKDL, that may have important implications for control of VL.

## Methods

### Study population

This is a hospital based retrospective study of PKDL cases diagnosed over last two decades, between January 1995 to December 2014, at Department of Dermatology, Safdarjung Hospital, New Delhi. A total of 282 cases from Bihar and its adjoining states namely Eastern Uttar Pradesh, West Bengal and Jharkhand, were registered. Majority of them belonged to economically underprivileged strata of society who migrated to Delhi for work where they were seen in tertiary hospital settings. Clinico-epidemiological details such as age, gender, nativity, history of VL, age at the time of VL, drug taken for VL treatment, history of PKDL and origins of PKDL cases were recorded for each individual at the time of reporting.

### Diagnosis

All patients were clinically examined for the identification of characteristic lesions such as macular, papular, nodular or mixed/polymorphic forms. They were examined for skin sensation and subjected to analysis by direct microscopy in skin tissue/slit aspirates sample. In later years, the cases were also subjected to rapid rK39 immuno-chromatographic strip test. Diagnosis was confirmed by demonstration of *Leishmania* amastigotes by microscopy and/or demonstration of *Leishmania* DNA with PCR/qPCR test as reported earlier [14, 15].

### Categories of PKDL based on clinical presentation

Confirmed cases of PKDL were categorized into four groups based on clinical presentation.

1. Macular PKDL: Cases presenting predominantly with hypopigmented lesions.
2. Papulonodular PKDL: Cases presenting papular and/or nodular lesions.
3. Mixed/polymorphic PKDL: Cases with all the three type of lesions i.e. macular, papular and nodular.
4. Others: Cases with unusual presentations viz. erythrodermic, fibroid type, plaque, ulcerated skin lesions.

### Treatment categories

1. Pre-miltefosine era: Majority of cases were treated with SAG monotherapy, at the dose of 20 mg/kg/day parenterally, either intramuscularly or intravenously, not exceeding a total daily dose of 1 g (10 ml) for a minimum period of 4 months or longer if necessary. Additionally, combination therapy with SAG was used in some cases. Four groups were formed in which SAG was given along with either allopurinol 800 mg/day in divided doses, rifampicin 15 mg/kg, or both allopurinol and rifampicin. Another group was given Mw vaccine (Immuvac, Cadila Pharmaceuticals, Ahmedabad, India), along with SAG, at the dose of 0.1 ml (containing heat-killed Mycobacterium w 0.5 × 10^9^ cells) intradermally at each site on the first visit, and repeated every fortnight to a total of five doses. The patients in all groups were seen every fortnight for the first month and later monthly, except those on vaccine therapy [16].
2. Miltefosine era: From 2009 onward, the cases were treated with oral miltefosine, 50 mg thrice daily for 2 months or twice daily for 3 months for adults [17] and 2.5 mg/kg/day for 3 months in children. Pregnant or lactating women and patients with systemic ailments including HIV cases were excluded. One case was exclusively treated with amphotericin B (i.v. 50 mg/day, total dosage of 4.5 g), with strict monitoring of renal functions [18]. The patients were followed-up every 6 months up to 18 months, whenever possible.

### Ethics statement

All the patients were diagnosed and treated as per the National Health Policy applicable at the time and following the guidelines of the Ethical Committee, VMMC & Safdarjung Hospital, New Delhi. The clinico-epidemiological details of patients were collected maintaining the confidentiality of the subjects under study.

### Statistical analysis

The data were analyzed using SPSS software (version 19.0, SPSS, Inc., Chicago, IL, USA). The strength of association between various variables and significance was determined using chi-square (*χ*2) test. *p* values < 0.05 were considered as significant.

## Results

### PKDL predominant in areas of high endemicity for VL

**Fig. 1:**
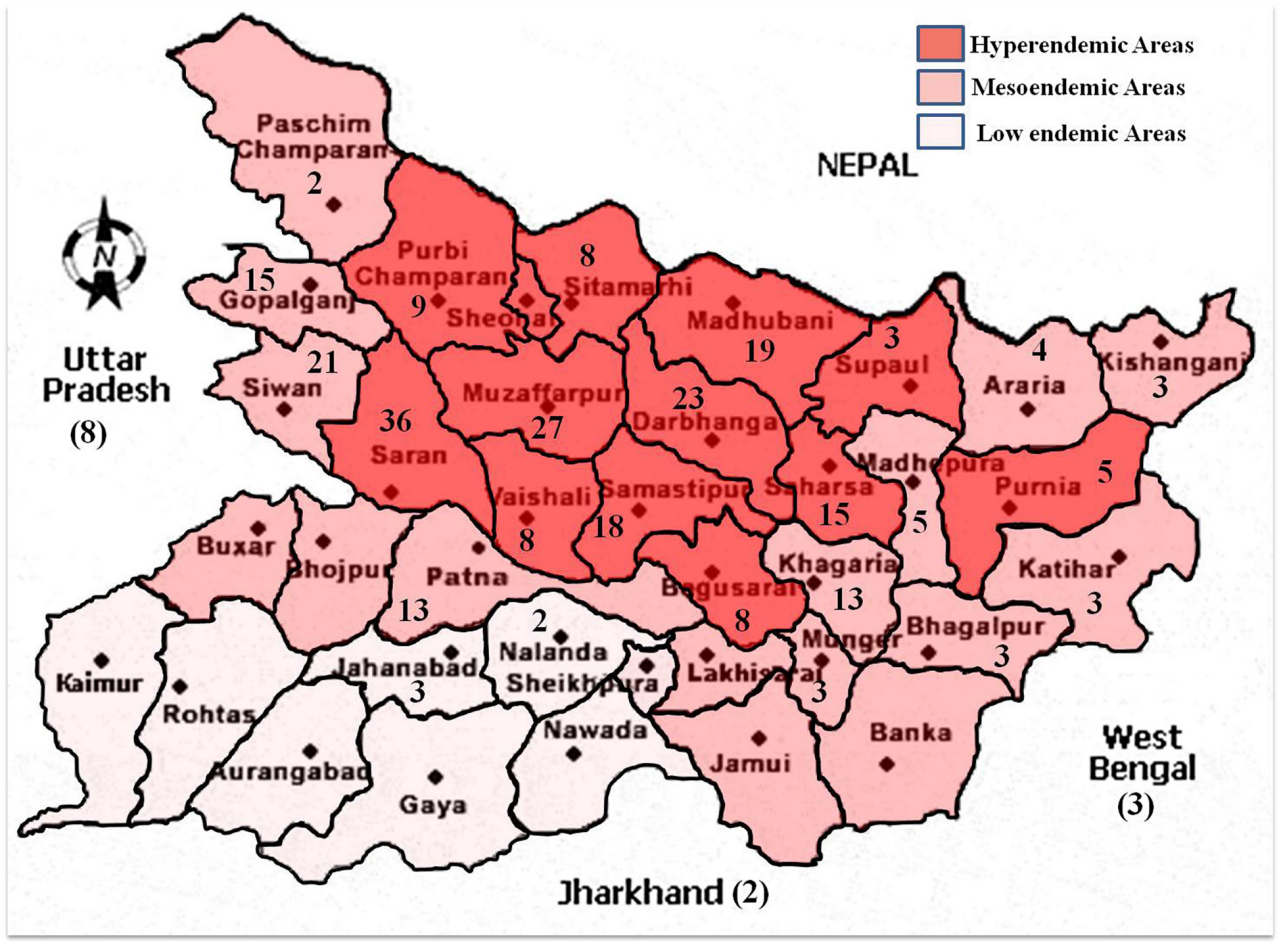
Distribution of PKDL cases in Bihar and the adjoining states. Map showing the distribution of PKDL cases in the state of Bihar and adjoining states, based on the area of high, moderate and low endemicity for VL, designated as per Sundar et al. [19]. Number shown in the figure is the number of PKDL cases from the district

**Fig. 2:**
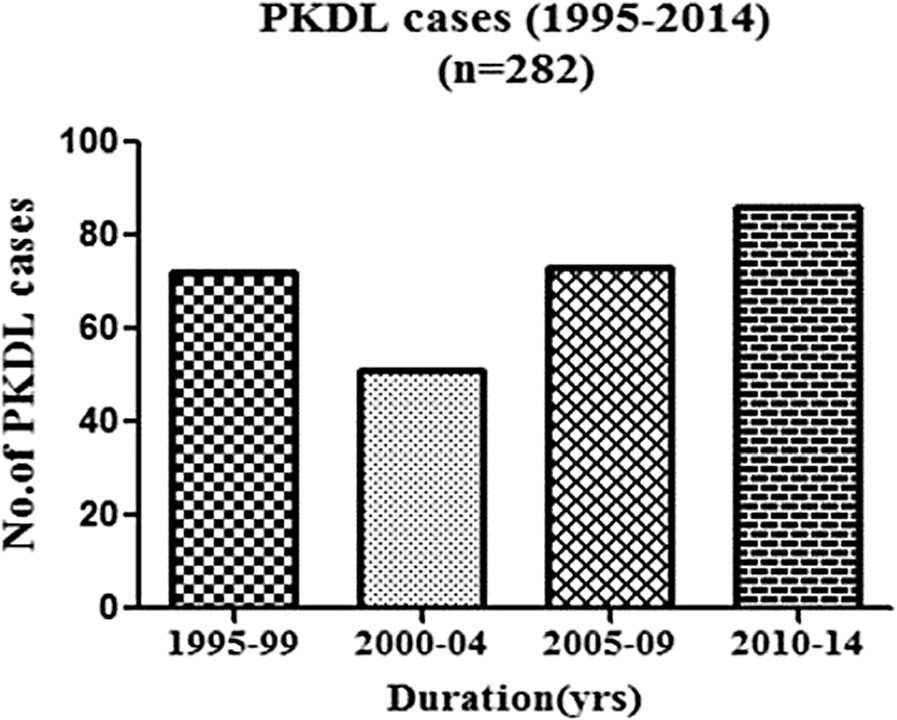
Incidence of PKDL in the reported period. Number of cases reported to Safdarjung Hospital, New Delhi, India per five year block duration since 1995

A total of 282 PKDL cases (Male, *n* = 225, Female, *n* = 57) were registered over last two decades since the year 1995 (Fig. 1). Majority (94.3 %, *n* = 266) of them originated from Bihar, and the rest (5.7 %, *n* = 16) were from the adjoining states namely Eastern Uttar Pradesh (3.5 %, *n* = 10), West Bengal (1.4 %, *n* = 4) and Jharkhand (0.7 %, n = 2). VL endemic areas in the state of Bihar have been categorised into high-, meso-, and low endemic areas based on the presence of degree of antimony resistance as reported earlier [19]. Adjoining states (Eastern Uttar Pradesh, West Bengal and Jharkhand) were categorised as low endemic region. Based on this classification, 63.5 % (*n* = 179) of PKDL cases originated from high endemic area as against 30.1 % (*n* = 85) and 6.4 % (*n* = 18) from meso- and low-endemic areas respectively (Fig. 1). We observed an upward trend in reporting of PKDL cases to Safdarjung Hospital, New Delhi, India since the year 2005 (Fig. 2).

### Clinico-epidemiological characteristics

Our study recorded 79.8 % (*n* = 225) male and 20.2 % (*n* = 57) female with overall median age of 22 years (range 5–65 years). The median age of females (20 years, range = 8–60) was comparable to that of males (22 years, range = 5–65, *p* = 0.789). Majority of cases belonged to the age group 19–44 years (64.5 %, *n* = 182), followed by paediatric cases aged ≤ 18 years (30.1 %, *n* = 85) and the group aged ≥ 45 years (5.3 %, *n* = 15).

Mixed/polymorphic form of lesions were predominant in 53.5 % cases (*n* = 151) followed by macular lesions (23.1 %, *n* = 65) and papulonodular lesions (21.6 %, *n* = 61). Unusual clinical lesions such as erythrodermic, fibroid type, plaque, ulcerated skin lesions were observed in 1.8 % (*n* = 5) cases. Polymorphic/mixed forms were predominantly present in both genders (53.3 % males, *n* = 120; 54.4 % females, *n* = 31) followed by papulonodular form in males (24.9 %, *n* = 56) and macular in females (36.8 %, *n* = 21). Papulonodular lesions were the least frequent in female (8.8 %, *n* = 5) cases whereas the macular lesions were the least frequent in males (19.5 %, *n* = 44). The unusual clinical lesions were exclusively observed in male cases (2.2 %, *n* = 5). There was a significant association between type of lesions and age (*χ*2 = 40.775 (6), *p* = 0.001), polymorphic/mixed lesions were predominant in adults aged between 19–44 years. Furthermore, type of clinical presentation were not associated with PKDL cases by the area of origin (*χ*^2^ = 3.960(6), *p* = 0.682). Besides, we observed mucosal lesions in 34 PKDL cases, all males. Majority of them (*n* = 33) received SAG treatment and one received amphotericin B treatment for VL. Mucosal involvement showed no association with lesion types (*χ*^2^ = 4.916(3), *p* = 0.178).

In the present study, 79.8 % (*n* = 225) of PKDL patients reported history of VL. Among PKDL patients with history of VL, 52.9 % (*n* = 119) had mixed/polymorphic lesions, 24.9 % (*n* = 56) had only macular lesions and 20.9 % (*n* = 47) had either papular and/or nodular. The remaining 1.3 % (*n* = 3) cases had unusual clinical presentations like erythrodermic, fibroid or plaque. There was no association between type of clinical presentation and history of VL (*χ*^2^ = 2.289 (3) *p* = 0.515). In addition, there was an evidence of association (*χ*2 = 9.681(2), *p =* 0.008) between cases with history of VL and the place of origin, indicating that the majority of the PKDL cases with history of VL originated from high endemic zones. Overall, the median time of manifestation of PKDL after VL treatment was 36 months (range = 1–384 months). PKDL lesions developed within 1 year in 13.3 % (*n* = 30), within 2–5 years in 56 % (*n* = 126) and after 5 years or more in 30.7 % (*n* = 69) cases, after apparent cure from VL (Table 1). Majority of cases with history of VL (93.3 %, *n* = 210) had been treated for VL with SAG while the remaining were treated with amphotericin B (5.3 %, *n* = 12) or miltefosine (1.3 %, *n* = 3). The median time lapse after VL treatment with SAG was 36 months (range, 1–384 months) which was less compared with amphotericin B (48 months, 3.6 – 84 months) but more compared to miltefosine treatment (21 months, range 12–36 months) (Table 2).

**Table 1:** Clinical presentation with respect to time lapse after VL

Time lapse after VL (yrs)	No. of macular lesions	No. of papulonodular lesions	No. of mixed lesions	No. of unusual clinical presentations	Total No. (%)	*p*
<1	9	3	18	0	30 (13.34)	*χ*2 = 13.324 (6), *p* = 0.038
1–5	33	30	63	0	126 (56)	
>5	13	15	38	3	69 (30.67)	
Total No. (%)	55 (24.44)	48 (21.33)	119 (52.88)	3 (1.34)	225

**Table 2:** Time lapse between VL and PKDL with respect to the drug used for VL treatment

Anti-leishmanial drug	No. (%) patients	Median time lapse after VL treatment (months)	Range (months)
SAG	210 (93.33)	36	1–384
Amphotericin B	12 (5.33)	48	3.6–84
Miltefosine	3 (1.33)	21	12–36

Besides, our data indicated that there was no association between type of drug used for VL treatment and the time lapse between VL treatment and PKDL incidence (*χ*2 = 1.994 (2), *p* = 0.369). We observed significant evidence of association (*χ*2 = 20.407(4), *p <* 0.001) between time lapse after VL treatment and age groups. The data indicated that majority of cases belonging to age group 19–44 yrs had onset of PKDL within 1–5 yrs post VL treatment. However, endemicity (*χ*2 = 0.332(4), *p* = 0.988) was independent of time lapse after VL treatment and onset of PKDL.

### Confirmatory diagnosis of PKDL based on minimally invasive sampling

**Fig. 3:**
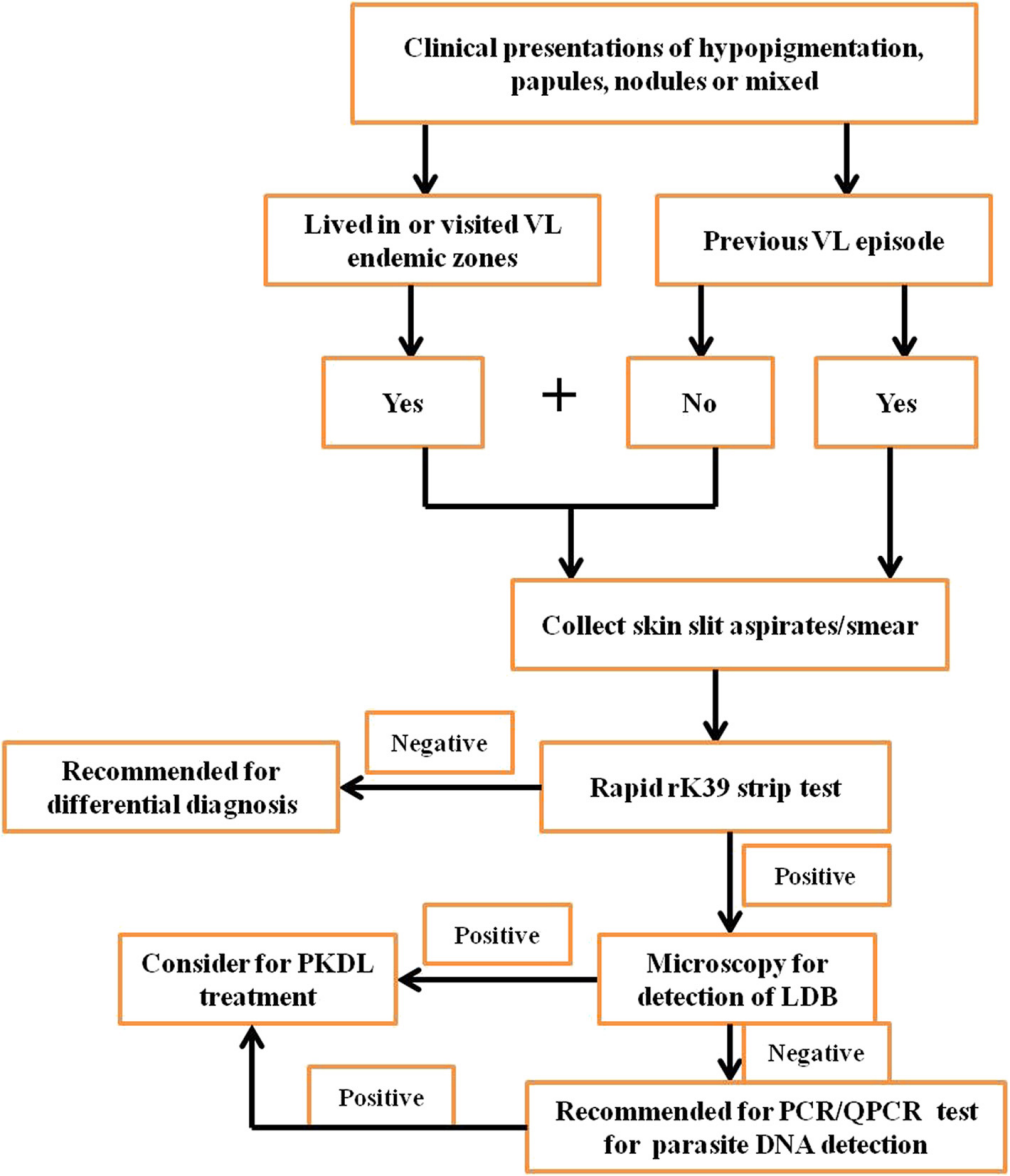
Diagnosis of PKDL based on minimally invasive sampling technique. The proposed flowchart for the confirmatory diagnosis of PKDL

In the present study, the median time between appearance of lesions and diagnosis was 30 months (range 1–240 months). Approx. 20.6 % (*n* = 57) of cases reported within 12 months and 54.9 % (*n* = 155) within 60 months after the appearance of lesions. Diagnostic sensitivity of microscopy for the demonstration of *Leishmania* amastigotes in two different samples such as tissue biopsy and skin slit smear was compared. The *Leishmania* amastigotes were evident in 31.5 % (89/282) tissue biopsy samples and in 36.2 % (107/282) slit aspirate samples. Diagnosis in cases negative by microscopy was confirmed by PCR/qPCR. We earlier demonstrated that rK39 strip test produced identical results with slit aspirate or serum as diagnostic sample for PKDL [20]. Furthermore, we reported skin slit aspirate as a good diagnostic specimen which offers sensitivity and specificity comparable to that of tissue biopsy with qPCR [20]. Therefore, we propose confirmatory diagnosis of PKDL using minimally invasive skin slit aspirate samples as described in Fig. 3.

Approximately, 26.95 % (*n* = 76) PKDL cases were initially misdiagnosed at primary health centers before they reported to our center, of which, 77.63 % (*n* = 59) cases had been misdiagnosed as cases of leprosy and received either complete or partial treatment for the same. The other misdiagnoses made were sarcoidosis in 11.84 % (*n* = 9) cases, secondary syphilis in 3.94 % (*n* = 3), rosacea in 3.94 % (*n* = 3) and pityriasis versicolor in 2.63 % (*n* = 2) cases.

### PKDL treatment

During the period from 1995 to 2008, a total of 59.57 % (*n* = 168) cases were diagnosed for PKDL and treated with SAG singly or in combination. Approximately 31.6 % (*n* = 89) PKDL cases were exclusively treated with SAG, of which only 14.6 % (*n* = 13) patients completed full treatment (Table 3). Combination of antimonials with weak antileishmanial drugs like allopurinol, rifampicin, allopurinol and rifampicin, Mw (Immunvac) were given in 79 cases. In the group receiving SAG with allopurinol (*n* = 56), only 17.8 % cases completed treatment and achieved cure; the corresponding figure in the group treated with SAG plus rifampicin group (*n* = 9) was 22.2 %. In SAG + rifampicin + allopurinol group (*n* = 6), none could continue injections beyond 1½ months. In the group where Mw vaccine was combined with SAG (*n* = 8), only 25 % adhered to complete treatment and attained cure [16].

**Table 3:** Outcome of treatment – compliance and relapse

Treatment	No. (%) patients	No. (%) of patients who completed treatment	No. (%) of relapse
SAG	89 (31.56)	13 (14.61)	-
SAG + Allopurinol	56 (19.86)	10 (17.85)	-
SAG + Rifampicin	9 (3.19)	2 (22.23)	Not followed
SAG + Allopurinol + Rifampicin	6 (2.12)	0 (0)	-
SAG + Mw Vaccine	8 (2.83)	2 (25)	-
Miltefosine	107 (37.94)	91 (85.05)	12 (13.18)
Amphotericin B	1 (0.35)	1 (100)	-
Miltefosine + Amphotericin B	6 (2.12)	5 (83.33)	-
Total	282	124 (43.97)	12 (9.67)

From 2009 onwards, a total of 113 patients were treated either with miltefosine alone (*n* = 107) or in combination with amphotericin B (*n* = 6). Of cases treated exclusively with miltefosine, 85 % (*n* = 91) completed treatment and achieved cure, while 14.9 % (*n* = 16) remained absconded. In the 18 months follow up period, we observed 13.2 % (*n* = 12) relapses (Table 3). Combination of miltefosine along with amphotericin B was given to 6 PKDL cases. One patient absconded and the remaining 5 completed treatment and none relapsed. One PKDL patient who was treated exclusively with amphotericin B remained cured.

## Discussion

This hospital based retrospective study over last two decades brings forth some essential points on clinical epidemiology of PKDL which would help establish appropriate control measures for VL. As expected, majority of PKDL cases originated from the area of high endemicity for VL, which could be due to multiple factors such as specific climatic conditions, socio-economic status, weak immune status, malnourishment [21]. There was an upward trend in the number of PKDL cases reporting to our center since the year 2005, probably due to higher awareness and increasing number of referrals to our center. With regard to clinical presentation, majority of cases had mixed/polymorphic form followed by macular and papulonodular lesions. Additionally, the study indicated a significant association between types of lesion with age, indicating that the polymorphic/mixed lesions were predominant in adults aged between 19–44 years. Our study revealed that there was mucosal involvement in 15 % cases, present across various lesion types. Mucosal invasion in PKDL is relatively uncommon and only sporadic cases have been reported [22]. A limitation of the current report is the selection of study population, that comprised mainly of migrants-for-work from endemic areas for VL, who were understandably predominantly males therefore gender based analysis were avoided.

Nearly one fifth of PKDL cases reported no history of VL. This could be because patients were either not aware of it or had asymptomatic infection in the past. The study revealed a significant association between time lapse after VL and the age group, indicating that cases in age group 19–44 year had onset of PKDL between 1–5 year following VL. However, the area of origin did not influence the time lapse between VL and PKDL. Nearly 70 % of cases manifested PKDL within 5 year following cure from VL, similar to an earlier report [12], however, this differs from the trend of PKDL development in Sudan where majority of cases develop lesions during VL or within the same year of VL treatment [23].

The variability of clinical presentations, poor awareness of this otherwise asymptomatic dermatosis, the migration of patients to non-endemic areas and lack of laboratory facilities to confirm diagnosis remain the major impediments in early recognition of PKDL [24]. In the present study, over 80 % cases were given a diagnosis after 12 months following first appearance of lesion, with overall median time lapse of 30 months. The delay in reporting could be explained as the disease is neither life threatening nor causing major discomfort and is common in people who mainly depend on daily wages for livelihood. Another reason for delayed diagnosis is misdiagnosis of the disease at various primary health centers, primarily as leprosy, a co-endemic dermatosis with high prevalence in the same areas. The early, accurate and feasible diagnosis of PKDL cases has been a long term goal that has important implications in the VL control program. Our data indicated that sensitivity of demonstration of *Leishmania* amastigotes by microscopy was 32 % to 36 % using slit smear or tissue biopsy, similar to the report from Bangladesh [25]. However, the sensitivity and specificity of molecular diagnostic methods such as PCR/qPCR was very high [14, 15]. These molecular tests gave equally reliable results using tissue sample or skin slit aspirates [20] although they have limitations such as high initial investment and high cost per test. We earlier demonstrated that rK39 strip test produced identical results with slit aspirate or serum as diagnostic sample for PKDL [20]. Furthermore, we reported skin slit aspirate as a good diagnostic specimen which offers sensitivity and specificity comparable to that of tissue biopsy with qPCR [20]. Therefore, we propose confirmatory diagnosis of PKDL using minimally invasive skin slit aspirate samples as described in Fig. 3. This procedure would reduce discomfort and permanent scarring and thus encourage patients to come forward for timely diagnosis and monitoring post-treatment.

During pre-miltefosine era, management of PKDL remained a challenge in terms of clinical diagnosis and treatment. In the present report, approximately one third PKDL cases were exclusively treated with SAG, of which only 14.6 % patients completed full treatment regimen (Table 3). The high volume of antimonials given parenterally, the long duration and the side effects could be the major impediments to this recommended therapy. Hence it was attempted to combine antimonials with weak antileishmanial drugs like allopurinol, rifampicin, allopurinol and rifampicin, Mw (Immunvac) to overcome these issues. Even with the combined regimen, duration of therapy with regular SAG injections did not significantly alter and hence there was no noticeable decrease in the overall dropout rate. Mw vaccine is derived from a nonpathogenic, saprophytic, Mycobacterium strain [26] and a chance observation of its efficacy in PKDL in the mid-nineties [4], led us to try it along with SAG. It is an immunomodulator for enhancing Th1 response of the host in many conditions. However, our experience with Mw vaccine in containing PKDL was less impressive. Similarly, Mw vaccine was also demonstrated to be ineffective in controlling experimental VL infection by *L. donovani* [27], contrary to its role in managing tuberculosis and leprosy infections. We observed that during pre-miltefosine era, over 85 % dropouts in PKDL cases across various treatment regimens were recorded and these partially treated cases were the potential reservoir of the *Leishmania* parasites especially in the endemic regions.

Another promising anti-leishmanial drug, amphotericin B also showed poor compliance since it required hospitalization. In recent times, single dose of liposomal form of amphotericin B such as Ambisome has been shown to be highly effective against both VL and PKDL [28–30]. Another antileishmanial drug, miltefosine was introduced in India in 2002, for the treatment of VL and was subsequently recommended for PKDL. This heralded the miltefosine era where the patients could take the drug orally and get cured. It proved to be a giant step in management of PKDL [31]. In the present report, a total of 107 cases of PKDL were exclusively treated with miltefosine, of which 85 % had successfully completed treatment regimen and achieved cure. In those patients who remained regular, mucosal lesions were the first to regress, followed by nodules while macular lesions took the longest time to show improvement. Initially, there were no relapses in one year follow-up however, in recent years there were 13.2 % relapses at 18 months follow-up, probably due to increase in tolerance to miltefosine. Hence, attempts were made to treat PKDL using combination of miltefosine and amphotericin B in a small number of cases which led to successful treatment. Similar to the antimony era where *Leishmania* parasites showed gradual increase in tolerance and resistance, miltefosine has also been reported to exhibit similar behavior [32]. This calls for formulations of newer and effective oral drugs which can be used singly or in combination to combat resistance.

## Conclusions

PKDL requires major attention for its proper management and thus control of VL. This hospital based study brings forth some essential points on trends in clinical and epidemiological features of PKDL cases. Higher incidence of PKDL was observed in areas of high endemicity for VL. Importantly, majority of the patients tend to delay reporting to primary health centers and are often misdiagnosed. Confirmatory diagnosis based on minimally invasive skin slit aspirate samples needs to be practised to minimize discomfort and scarring, thus encouraging the patients to come forward for early diagnosis as well as follow up. Furthermore, there was a dramatic increase in compliance with oral miltefosine treatment; however increasing relapse rate in long term follow up warrants the introduction of newer/combination therapies with oral formulations.
